# Evaluation of chemotherapy and P2Et extract combination in ex-vivo derived tumor mammospheres from breast cancer patients

**DOI:** 10.1038/s41598-020-76619-9

**Published:** 2020-11-12

**Authors:** Claudia Urueña, Tito A. Sandoval, Paola Lasso, Mauricio Tawil, Alfonso Barreto, Lilian Torregrosa, Susana Fiorentino

**Affiliations:** 1 Grupo de Inmunobiología y Biología Celular, Unidad de Investigación en Ciencias Biomédicas, Facultad de Ciencias, Pontificia Universidad Javeriana, Carrera 7a. No. 43-82, Ed. 50, Lab. 101, 110211 Bogotá, Colombia; 2 Hospital Universitario San Ignacio, Centro Javeriano de Oncología, Facultad de Medicina, Pontificia Universidad Javeriana, Bogotá, Colombia

**Keywords:** Breast cancer, Cancer stem cells, Cancer therapy

## Abstract

The main cause of death by cancer is metastasis rather than local complications of primary tumors. Recent studies suggest that breast cancer stem cells (BCSCs), retains the ability to self-renew and differentiate to repopulate the entire tumor, also, they have been associated with resistance to chemotherapy and tumor recurrence, even after tumor resection. Chemotherapy has been implicated in the induction of resistant phenotypes with highly metastatic potential. Naturally occurring compounds, especially phytochemicals such as P2Et, can target different populations of cancer cells as well as BCSC, favoring the activation of immune response via immunogenic tumor death. Here, we evaluated the presence of BCSC as well as markers related to drug resistance in tumors obtained from 78 patients who had received (or not) chemotherapy before surgery. We evaluated the ex vivo response of patient tumor-derived organoids (or mammospheres) to chemotherapy alone or in combination with P2Et. A xenotransplant model engrafted with MDA-MB-468 was used to evaluate in vivo the activity of P2Et, in this model P2Et delay tumor growth. We show that patients with luminal and TNBC, and those who received neoadjuvant therapy before surgery have a higher frequency of BCSC. Further, the treatment with P2Et in mammospheres and human breast cancer cell lines improve the in vitro tumor death and decrease its viability and proliferation together with the release of immunogenic signals. P2Et could be a good co-adjuvant in antitumor therapy in patients, retarding the tumor growth by enabling the activation of the immune response.

## Introduction

According to the World Health Organization (WHO), for 2018, at least 18 millions of new cases of cancer were diagnosed, and around 9 millions of deaths for these group of diseases. For breast cancer, the statistics pointed for at least 2.1 million (11.6%) of new diagnosed cases and 626,679 (6.6%) of deaths for the same year, ranking the breast cancer as the one with the highest mortality in the world^1^. Breast cancer is classified in: Luminal A, Luminal B, Basal, ErbB2- overexpressing, and Normal breast–like, with differences in 5-year survival rate^2^. A new classification based on integrated analysis of both genomic and transcriptomic data from primary breast tumors, METABRIC (MolEcular TAxonomy of BReast cancer International Consortium)^2^, revealed 10 integrative clusters with distinct clinical outcome^3^. This diversity is also observed inside triple-negative breast cancer (TNBC)^4^.

The main cause of death by cancer is metastasis, and a significant number of cases remain refractory and relapse is commonly observed. The cumulative rate of distant metastasis was 44.0% after 22 years of follow-up^5^. In this regard, recent studies have suggested that a small population of cells termed breast cancer stem cells (BCSCs), retain the ability to self-renew and differentiate to repopulate the entire tumor^6^. Further, BCSCs have been associated with resistance to chemotherapy as well as radiotherapy and recurrence, even after tumor resection^6^. In recent years, big efforts were made to establish the signature features that define the BCSC population; surface markers like CD44 combined with CD24, CD49f. or CD133, detoxifying enzymes like Aldehyde dehydrogenase (ALDH1^high^), epithelial specific antigen (EpCAM or ESA), ABCG2^7^, and the expression of Sox2, Oct4 and Nanog transcription factors^8,9^, have been used.

ALDH is one of the most important features related to resistance in cancer stem cells (CSCs)^7^. ALDH^high^ tumor cells are more resistant to treatment with radiation and drugs, such as gentamycin, carboplatin, etoposide and paclitaxel^10^. ALDH^high^ CSCs seems to be involved in invasive and metastatic behavior in inflammatory breast cancer, and their presence in the tumor tissue of patients is a prognostic marker to predict metastasis and poor patient outcomes^6^. On the other hand, CD49f has been correlated with taxanes resistance principally in TNBC^11^. Drug resistance in BCSCs have been principally attributed to the increased expression of multidrug resistance (MDR) transporters including ATP-binding cassette (ABC) efflux pumps as P-glycoprotein (P-gp/*ABCB1*), multidrug resistance-associated protein 1 (MRP1/*ABCC1*) and breast cancer resistance protein (BCRP/*ABCG2*)^7^. Further, CSC are difficult to target due to features like an OXPHOS-like metabolism, a quiescent state that dependent of the niche, among others^12^.

Chemotherapy has been implicated in the induction of resistant phenotypes with highly metastatic potential, but the mechanisms involved are not entirely clear^13^. It was recently demonstrated that taxanes and anthracyclines, cause tumor-derived extracellular vesicles with enhanced pro-metastatic ability^14^. Also, exposure of breast cancer cells lines to chemotherapy leads to an enrichment of BCSCs by the induction of glutathione *S*-transferase omega 1 (GSTO1), the cystine transporter xCT and the regulatory subunit of glutamate-cysteine ligase (GCLM) dependent on hypoxia-inducible factor 1 (HIF-1) and HIF-2. Together, these mechanisms induce STAT3 signaling activation, implicated in the expression of pluripotency factors, BCSC enrichment and immunosuppressive remodeling of the tumor microenvironment^15,16^.

Although chemotherapy may be pro-metastatic, it may also favor the activation of the immune response. The immune system plays a dual role in breast cancer since it promotes tumorigenesis^17^, but also participates in the elimination of BCSC^18^. The long-term success of cancer therapy is related in part to the ability to decrease the suppressive microenvironment of the tumor and activate the immune response by a process called immunogenic cell death, characterized by the expression of calreticulin, HMGB1 and ATP release, to further activate immune recognition and killing of the tumor cells^19^. Phytochemicals exhibit the ability to target heterogeneous populations of cancer cells as well as CSCs^20^. Previously, our group identified a gallotannin-rich extract from *Caesalpinia spinosa* (P2Et) with tumoricidal effects in breast and melanoma murine models^21,22^. This extract contains galloylquinic acid derivatives in high proportions as well as pentagalloylglucose and other gallic acid-containing compounds (gallates) in lower proportions^21,23^. P2Et act as a potent antioxidant and is highly cytotoxic against tumor cells, in particular those expressing drug resistance pumps^23^. Furthermore, the anti-tumor activity of P2Et requires an intact adaptive immune system in the B16F10 melanoma model^22^. We have evaluated the effects of P2Et in breast ALDH^+^-tumor models (in vitro and in vivo), and we found the presence of cytotoxic T cells capable of lysing both the 4T1 and 4T1-ALDH^+^ cells, providing evidence about the role of the immune response in the control of CSCs^24^. In the present study, we evaluated the presence of BCSC as well as markers related to drug resistance in tumors obtained from patients who had received (or not) debulking neoadjuvant therapy before surgery. Then, we evaluated the ex-vivo response of mammospheres derived from these samples to conventional chemotherapy alone or in combination with P2Et. Finally, we tested P2Et in a xenotransplant model of triple-negative (TN) human tumor cells.

The results suggested that patients with luminal and TNBC, as well as patients who received neoadjuvant therapy before surgery have a higher frequency of BSCS. Further, the treatment with P2Et in tumor-derived organoids and human breast cancer cell lines induced the ex vivo tumor death together with the release of immunogenic signals, suggesting that P2Et could favor the activation of the immune response and could be used as co-adjuvant for antitumor therapy in breast cancer patients.

## Results

### Luminal and triple negative patients have a higher frequency of breast cancer stem cells (BCSC)

78 breast cancer (BC) patients and 7 healthy donors (HD) were evaluated for the frequency of BCSC, CD45^+^ cells, and multidrug efflux pumps expression (BCRP, Pgp and MRP1). Patients’ age was ranged from 30 to 92 years; mean age at diagnosis was 61.3 ± 1.3 years. The number of patients in stage I were (n = 9), stage II (n = 34), stage III (n = 31) and stage IV (n = 3). The estrogen receptor (ER), progesterone receptor (PR), *Her2* expression and Ki-67 percentage, were used to classify the samples, as follows: Luminal A (n = 17), Luminal B (n = 42), Triple negative (TN) (n = 14) and *Her2* (n = 5). Additionally, 27 patients received neoadjuvant chemotherapy (NAT) before surgery and 51 patients did not receive NAT. Received NAT regimens are shown in Table 1.

**Table 1:** Clinicopathological characteristics of patients with breast cancer.

Characteristics	Luminal A (n = 17)	Luminal B (n = 42)	Triple negative (n = 14)	Her2 (n = 5)
**Age (years)**				
< 40	0 (0)	2 (4.8)	3 (21.4)	0 (0)
40–49	1(5.9)	5 (11.9)	2 (14.3)	1 (20.0)
50–65	7 (41.2)	18 (42.9)	7 (50)	1 (20.0)
> 65	9 (52.9)	17 (40.5)	2 (14.3)	3 (60.0)
**Lymph nodes**				
Negative	12 (70.6)	19 (45.2)	8 (57.1)	2 (40.0)
Positive	3 (17.6)	22 (52.4)	6 (42.9)	2 (40.0)
Unknown	2 (11.8)	1 (2.4)	0 (0)	1 (20.0)
**TNM State (AJCC)**				
I	2 (11.8)	6 (14.3)	1 (7.1)	0 (0)
II	14 (82.4)	16 (38.1)	3 (21.4)	1 (20.0)
III	0 (0)	18 (42.9)	10 (71.4)	3 (60.0)
IV	0 (0)	2 (4.8)	0 (0)	1 (20.0)
Unknown	1 (5.9)	0 (0)	0 (0)	0 (0)
**ER**				
Negative	0 (0)	0 (0)	14 (100)	5 (100)
Positive	17 (100)	42 (0)	0 (0)	0 (0)
**PR**				
Negative	0 (0)	8 (19.0)	14 (100)	5 (100)
Positive	17 (100)	34 (81.0)	0 (0)	0 (0)
**HER2**				
Negative	16 (94.1)	33 (78.6)	14 (100)	0 (0)
Positive	1 (5.9)	9 (21.4)	0 (0)	5 (100)
**Ki67**				
< 15%	17 (100)	1 (2.4)	0 (0)	1 (20.0)
> 15%	0 (0)	41 (97.6)	13 (92.9)	3 (60.0)
Unknown	0 (0)	0	1 (7.1)	1 (20.0)
**NAT before surgery**				
No NAT	14 (82.4)	32 (76.2)	4 (28.6)	1 (20.0)
AC	2 (11.8)	0 (0)	2 (14.3)	1 (20.0)
TX	0 (0)	2 (4.8)	1 (7.1)	3 (60.0)
AC + TX	1 (5.9)	8 (19.0)	6 (42.9)	0 (0)
FEC + TX	0(0)	0 (0)	1 (7.1)	0 (0)

*ER* estrogen receptor; *PR* progesterone receptor; *HER2* human epidermal growth factor receptor 2; *NAT* neoadjuvant chemotherapy; *AC* anthracyclines; *TX* taxane; *FEC* 5-fluoroacil, epirubicin, cyclophosphamide.

**Figure 1:**
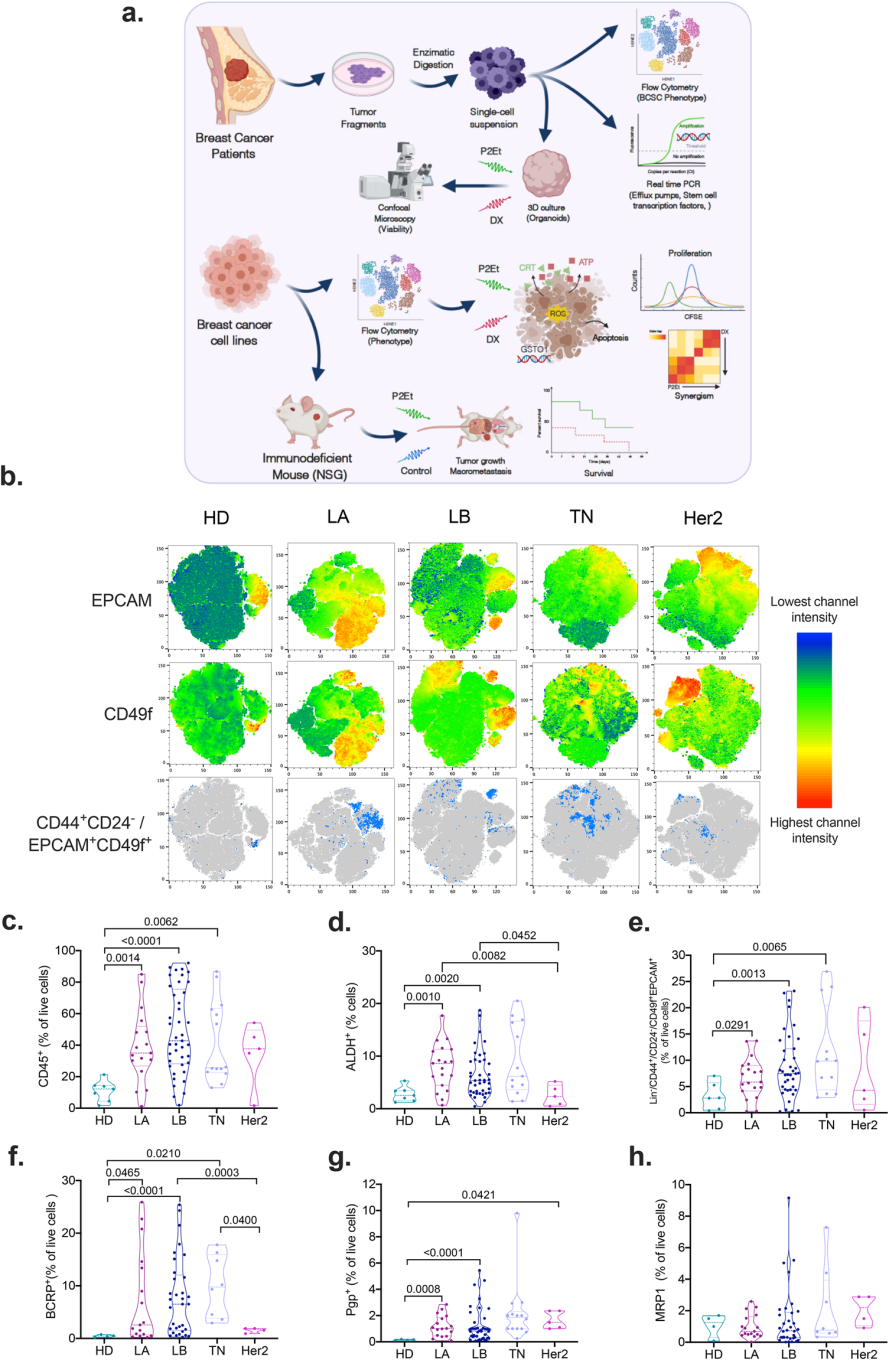
Cancer stem cell features in breast cancer patients. The patients were classified in Healthy Donor (HD), Luminal A (LA), Luminal B (LB), Triple negative (TN) and Her-2+. (**a**) Sample processing workflow of breast cancer tumors. (**b**) Multidimensional reduction analysis (tSNE) of flow cytometry data by breast cancer sub-type, rainbow scale represents relative intensity by channel; bulk population (gray) and CD44 + CD24-EPCAM + CD49f + (light blue). (**c**) CD45 + tumor-infiltration lymphocytes. (**d**) Frequency of Aldehyde dehydrogenase 1 (ALDH1) positive cells. (**e**) Frequency of Lin-CD44 + CD24-CD49f + EPCAM + cells. (**f**) Frequency of BCRP+. (**g**) Frequency of Pgp+. (**h**) Frequency of MRP1 + cells by flow cytometry. Data are presented as violin plots and each point represents independent samples; dotted lines indicate quartiles. Multiple comparisons were calculated by one-way ANOVA with Dunnet T3 correction, and significant exact *p*-values are shown. *p < 0.05; **p < 0.01; ***p < 0.001, **** p < 0.0001.

The BCSC characterization was performed using primary tumor tissue collected during the resection surgery and processed as shown in Fig. 1a. Single cell suspensions were obtained by mechanical and enzymatic digestion as previously described^25^ (Fig. 1a), and tumor-derived organoids (mammospheres) were generated, this system provide higher physiological relevance than 2D-cultures, allowing the propagation of mammary stem and progenitor cells. The cells were analyzed by flow-cytometry excluding lineage negative populations (CD45, CD140b, CD31), staining for putative BCSC markers (CD24, CD44, EPCAM and CD49f) and multidrug efflux pumps (BCRP, Pgp, MRP-1). Additionally, we measured the ALDH enzymatic activity.

Multi-dimensional reduction analysis revealed the expression of putative BCSC markers (Fig. 1b). Due to different acquisition dates it was impossible to generate a common tSNE plot for all the samples; however, the BCSC (CD44^+^CD24^−^EPCAM^+^CD49f^+^) can be appreciated (Fig. 1b). The BCSC characterization was performed as shown in Supplementary Fig. S1a,b. Further analysis showed high infiltration of CD45^+^ cells in Luminal and TN patients compared with HD (Fig. 1c). Higher frequency of BCSC ALDH^+^ or Lin^-^CD44^+^CD24^-^CD49f.^+^EPCAM^+^ phenotype was evident in Luminal and TN patients (Fig. 1d,e), together with overexpression of BCRP^+^ and Pgp^+^ but not MRP1^+^ as compared with HD (Fig. 1f–h). In accordance with our data, a recent study showed the elevated expression of ALDH1 in a cohort of breast cancer patients in TN samples measured by IHC^26^. The expression of *ABCG2*, *ABCB1*, *ABCC1* and *Nanog*, *Sox2* and *Oct4* was evaluated by real time PCR, but differences were observed as compared with HD (Supplementary Fig. S2).

### Neoadjuvant chemotherapy (NAT) promotes enrichment of the BSCS into the tumor tissue

**Figure 2:**
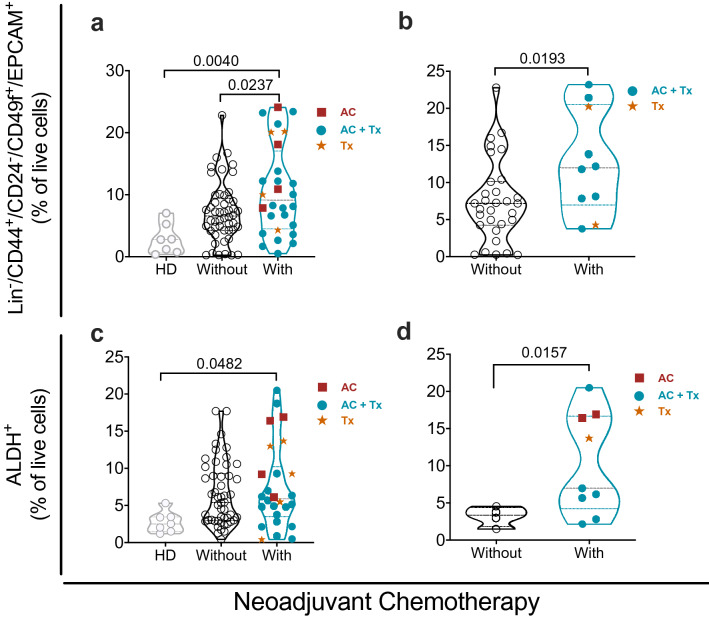
Neoadjuvant chemotherapy (NAT) before surgery increase the frequency of BCSC. (**a**) Frequency of BCSC (Lin-/CD44 + CD24-CD49f + EPCAM+) in healthy donor (HD), breast cancer patients with or without NAT before surgery. (**b**) Frequency of BCSC in LB patients with or without NAT before surgery. (**c**) Frequency of BCSC (ALDH +) in HD, breast cancer patients with or without NAT before surgery. (**d**) Frequency of BCSC (ALDH +) in TN breast cancer patients who received or not NAT before surgery. (AC (Anthracyclines + Ciclophosphamide), AC + TX (Anthracyclines/Ciclophosphamide + Taxanes), TX (Taxanes). Data are presented as violin plots and each point represents an independent sample; dotted lines indicate quartiles. Multiple comparisons were calculated by one-way ANOVA with Tukey post-test analysis and unpaired t-test, significant exact *p*-value are shown. *p < 0.05; **p < 0.01.

**Figure 3:**
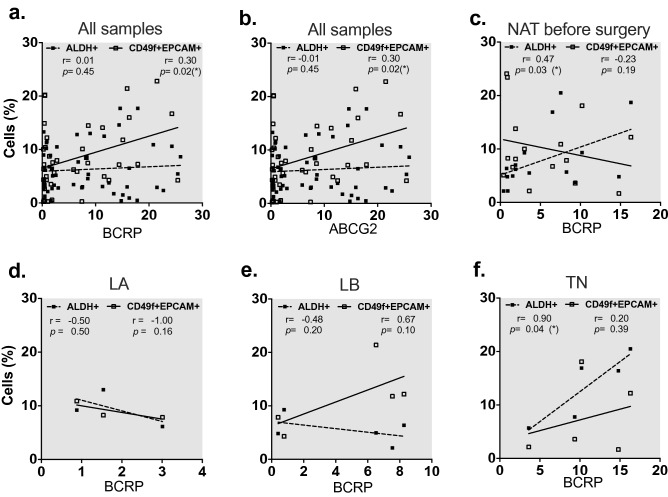
Breast cancer stem cells markers (ALDH+) correlated with BCRP expression in triple negative patients. Correlation of BCRP protein and ABCG2 gene expression with BCSC markers (ALDH+ and CD24-CD44 + CD49f + EPCAM +) in breast cancer patients who received or not NAT before surgery. (**a**) Correlation of BCRP protein with ALDH + and CD24-CD44 + CD49f+ EPCAM + expression in all samples. (**b**) Correlation of ABCG2 gene expression with ALDH + and CD24-CD44 + CD49f + EPCAM + in all samples. (**c**) Correlation of BCRP with ALDH + and CD24-CD44 + CD49f + EPCAM + expression in breast cancer patients who received NAT before surgery. Correlation of BCRP protein with BCSC markers (ALDH+ and CD24-CD44+ CD49f + EPCAM+) expression in (**d**) LA only, (**e**) LB only and (**f**) TN patients who received NAT before surgery. Correlations were assessed using nonparametric Spearman correlation, determination coefficient r and *p*-value are shown. *p < 0.05.

It has been extensively documented that neoadjuvant therapy impact in the frequency of CSC^27^. We interrogated whether NAT impacted the frequency of BCSC into the tumor tissue of patients who received (or not) treatment before surgery. As expected, NAT before surgery induce a higher frequency of Lin^-^CD44^+^CD24^−^CD49f.^+^EPCAM^+^ (BCSC) cells in comparison with the HD or the group of patients who had not received NAT (Fig. 2a). The highest statistically significant frequency was observed for Luminal B patients (Fig. 2b) compared to the other groups (Supplementary Fig. S3). On the other hand, we interrogated the presence of ALDH^+^ cells (BCSC) and significant differences were only observed between patients who received NAT before surgery and HD (Fig. 2c and Supplementary Fig. S3), being more evident in TN samples (Fig. 2d). We interrogated the correlation between BCSC and drug resistance, a positive correlation between BSCS markers (Lin^-^CD44^+^CD24^−^CD49f^+^EPCAM^+^, r = 0.30 *p* = 0.02) and BCRP protein (Fig. 3a) or *ABCG2* gene expression (Fig. 3b) was observed, regardless of the NAT therapy status.

Further, a positive correlation between BCRP1 and ALDH was found in patients with NAT before surgery (Fig. 3c), suggesting that ALDH expression is usually accompanied with other chemo-resistant traits as efflux pumps as previously reported^28^. Likewise, we found a positive correlation between ALDH and *ABCC1* gene expression (MRP1 pump) in the NAT group (Supplementary Fig. S4d), but in non-NAT treated group (Supplementary Fig. S4e,f). Also, a positive correlation between ALDH and BCRP expression in the group of TN patients was observed (Fig. 3d–f). Together, these results suggested that cells with a highly resistant phenotype could be selected in the tumors of patients after NAT, and these population was particularly high on TN patients, similar results were showed before^29^.

### P2Et extract decrease the viability of mammospheres derived from breast cancer patients after in vitro treatment

We have previously shown that P2Et induces immunogenic tumor cell death, with the consequent activation of adaptive immune response^21,22^. Moreover, P2Et inhibits sphere-formation of 4T1 ALDH^+^ CSC, and act synergistically with DX, in vitro and in vivo^23^.

**Figure 4:**
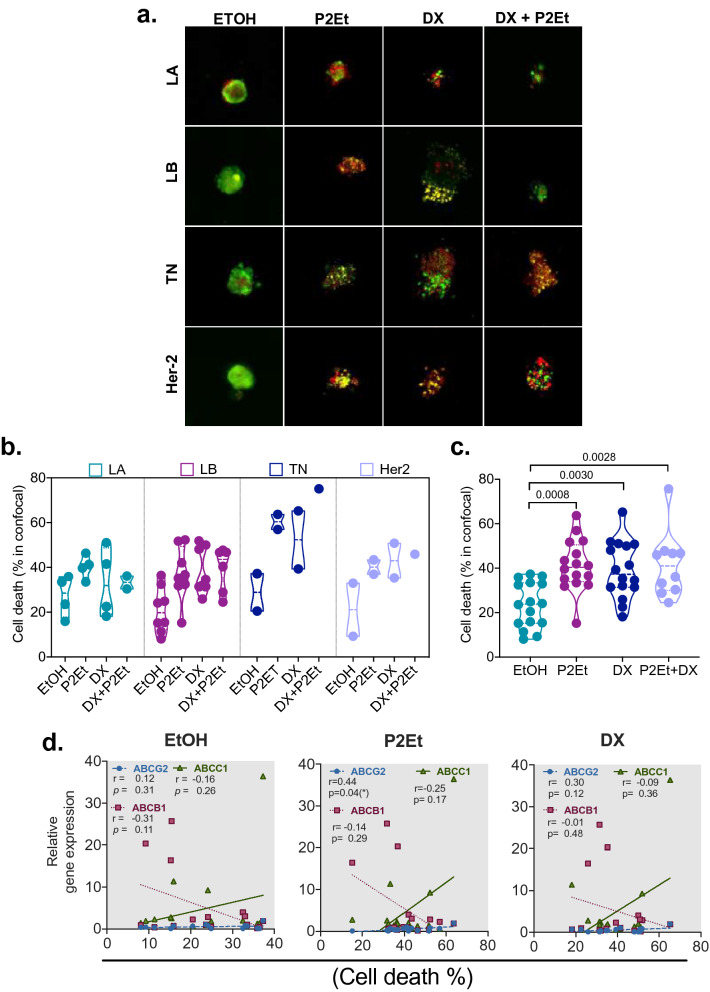
P2Et extract decrease the viability of mammospheres derived from breast cancer patients after in vitro treatment. (**a**) Representative confocal microscopy image of the effect of P2Et extract, DX or DX + P2Et on the mammospheres viability of breast cancer patients. Luminal A (LA), Luminal B (LB), Triple negative (TN) and Her2. Calcein AM (green), Ethidium homodimer-1 (Red). (**b**) Cell death percentage in mammospheres by molecular subgroups of breast cancer patients after treatment. (**c**) Cell death percentage in all mammospheres from breast cancer patients after treatment. Data are presented as violin plots and each point represents an independent sample; dotted lines indicate quartiles. Exact *p*-values were calculated using One-way ANOVA with Tukey post-test analysis. (**d**) Spearman nonparametric correlation between percentage of cell death with ABCB1, ABCG2, ABCC1 gene in mammospheres from breast cancer patients after P2Et, DX or Ethanol (negative control) treatment, coefficient of determination r and exact *p*-value are shown. *p < 0.05; **p < 0.01; ***p < 0.001.

With this on mind, we interrogated the effect of P2Et, DX or the combination of both (P2Et + DX) on mammospheres generated from patient samples in 3D cultures (Fig. 4a). Using this setting, we observed that mammospheres are more sensitive to P2Et treatment compared to the control group (Ethanol, negative control), independently of the molecular subtype; however, a trend to increased sensitivity was observed in tumor cells from TN patients (Fig. 4b). All the treatments induced significant cell death in 3D-culture conditions in comparison with vehicle (Fig. 4c).

Then, a positive correlation between the percentage of dead cells induced by P2Et and the gene expression of *ABCG2* was found (Fig. 4d) and not with the other variables studied (Supplementary Fig. S5). These findings confirm our previous results^23^. Thus, we observed that the human-derived mammospheres are sensitive to P2Et extract, and the effect was independent of the molecular subtype.

### P2Et extract decrease the viability and proliferation of human triple negative breast cancer cells

**Figure 5:**
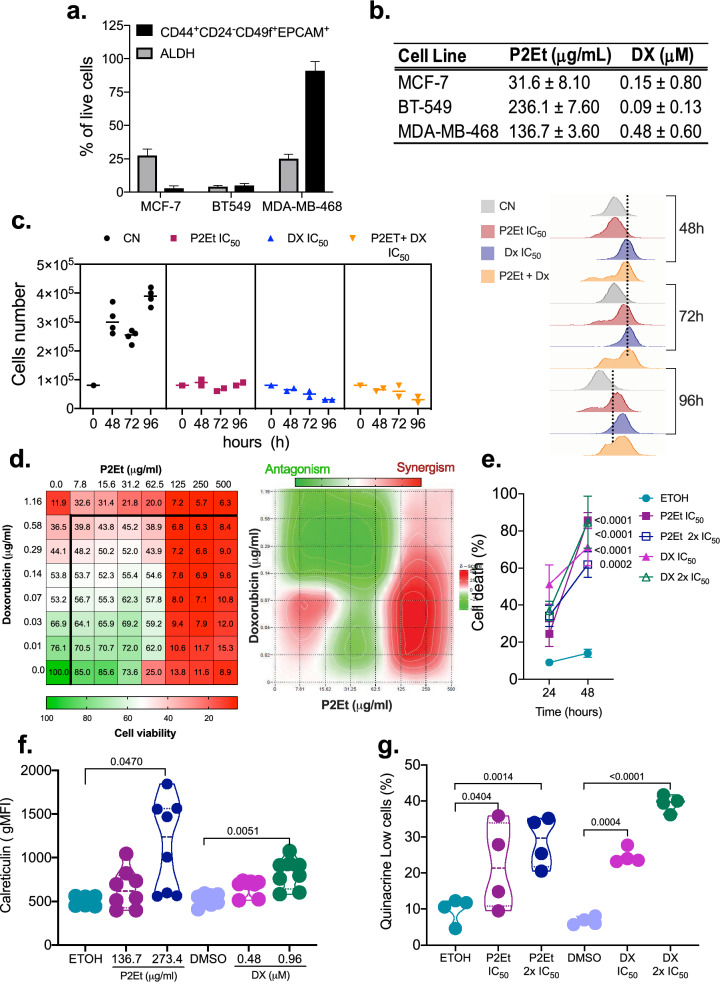
P2Et extract decrease the viability and proliferation of human triple negative breast cancer cells. (**a**) Frequency of BCSC ALDH1 + and Lin-CD44 + CD24-CD49f + EPCAM + cells in MCF-7, BT-549 and MDA-MB-468 breast cancer cells measured by flow cytometry. Data are presented as the mean ± SEM. (**b**) IC_50_ values of P2Et extract or Doxorubicin (DX) in MCF-7, BT-549 and MDA-MB-468 using MTT Assay. (**c**) Left side: MDA-MB-468 cell count by trypan blue after treatment with vehicle (Ethanol), P2Et IC50, DX IC50, or P2Et + DX IC_50_ for 0, 48, 72 and 96 h. Right side: MDA-MB-468 cells were stained with CFSE and treated with P2Et IC_50_, DX IC_50_, or P2Et + DX IC_50_ for 0, 48, 72 and 96 h. For each time the proliferation was evaluated by flow cytometry. (**d**) The combined inhibitory effects of P2Et (0 to 500 μg/ml) and DX (0 to 1.16 μg/ml) were tested over a range of combinations against MDA-MB-468 cells using MTT Assay (Left side); a dose–response matrix was generated and analyzed for zero-interaction potency method (ZIP), using SynergyFinder pipeline (Right side). Data are presented as the mean of three independent experiments. (**e**) Frequency of death (Annexin V+ , propidium iodide [PI]−) and Annexin V+ , PI+) MDA-MB-468 cells after 24 and 48 h of treatment with Etanol, P2Et (IC_50_ and IC_50_*2) or DX (IC_50_ and IC_50_*2). MDA-MB-468 cells were stained with Annexin V-Alexa Fluor 488 and PI. Data are presented as the mean value ± SEM of 4 independent experiments. (**f**) MDA-MB-468 cells were treated with vehicle (ethanol or DMSO), P2Et (IC_50_ and IC_50_*2) or DX (IC_50_ and IC_50_*2) for 24 h. Surface exposure of Calreticuline (CRT) was determined by flow cytometry among viable cells (Aqua negative). Data are presented as violin plots and each point represents independent samples; dotted lines indicate quartiles. (**g**) MDA-MB-468 cells were treated vehicle (ethanol or DMSO), P2Et (IC_50_ and IC_50_*2) or DX (IC_50_ and IC_50_*2) for 48 h. After treatment cells were stained with quinacrine (1 μM) and PI. Quinacrine low cells were determined among viable cells (PI negative cells). Data are presented as violin plots and each point represents independent samples; dotted lines indicate quartiles. Exact significant *p*-values are shown. *p < 0.05; **p < 0.01; ***p < 0.001, **** p < 0.0001.

TNBC represent 15–20% of all breast cancer cases^30^. Then, we explored the effect of P2Et in an in vivo model of triple negative human cells enriched in BCSC. First, we evaluated the ALDH activity, the expression of BCSC genes *(Nanog, Sox2* and *Oct4*) (Supplementary Fig. S6), multiresistance pumps (*ABCG2, ABCC1* and *ABCB1*) (Supplementary Fig. S6) and the BCSC frequency (CD44^+^CD24^+^CD49^+^EPCAM^+^) (Supplementary Fig. S1c,d) in three human breast cancer cell lines. MCF-7 (ER^+^) and MDA-MB-468 (TN) had the highest ALDH activity, 28 and 25%, respectively (Fig. 5a). The cell line MDA-MB-468 had the highest frequency of BCSC cells (Fig. 5a). The results suggested that MDA-MB-468 was the most sensitive to P2Et treatment, with an IC_50_ of 136.7 μg/mL compared with 236.1 μg /mL for BT-594 cell line (Fig. 5b). Also, we observed lower cell number (48, 72 and 96 h), and decreased proliferation ability of MDA-MB-468 cells upon the treatment with P2Et (Fig. 5c, Supplementary Fig. S7).

Further, we interrogate the synergistic effects of P2Et extract with DX, we performed MTT assays over several combinations and a synergistic effect was observed using higher concentrations of P2Et and lower concentrations of DX (Fig. 5d). It has been showed that chemotherapy induces the expression of glutathione S-transferase omega 1 (GSTO1) and further knockdown of GSTO1 expression, abrogates carboplatin induced BCSC enrichment, decreases tumor initiation, metastatic ability, and delays tumor recurrence after chemotherapy^15^. In light of this, we evaluated the effect of P2Et extract in the ROS and GSTO-1 gene expression; as anticipated, compared with DX, lower ROS production was observed upon P2Et in MDA-MB-468 cell line at 6, 12 and 24 h, while both treatments decreased GSTO-1 relative gene expression at 24, 48 and 72 h (Supplementary Fig. S8). These results could suggest that P2Et and DX induced cell death by different routes, implicating a different management of intracellular ROS, independent of glutathione.

Thus, we examined whether P2Et extract induce DAMPs in MDA-MB-468 cells. It was found that P2Et induced apoptosis (phosphatidyl serine externalization) (Fig. 5e) and CRT expression (Fig. 5f), which is significantly higher compared with cells treated with the ETOH (negative control) or DX. ATP release, another feature of immunogenic cell death, was observed upon both treatments (Fig. 5g), similar results were observed previously for DX and P2Et^31^ in other models of cancer.

### P2Et extract delay tumor growth in triple negative human breast cancer

**Figure 6:**
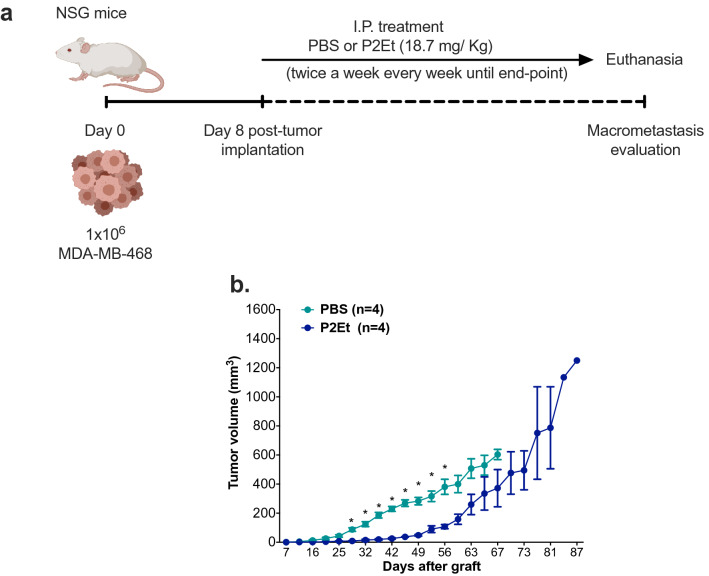
P2Et extract retard initially primary tumor growth but increase survival in triple negative human breast cancer. (**a**) Experimental scheme of treatment. MDA-MB-468 cells were inoculated into the mouse mammary gland, 8 days later, the mice were treated with PBS (negative control) or 18.7 mg/Kg of P2Et extract twice a week until endpoint. (**b**) Tumor growth (mm^3^) of MDA-MB-468 control (PBS) mice or mice treated with P2Et extract. Multiple t-test with Holm-Sidak method correction was performed assuming significance of α = 0.05. *p < 0.05.

MDA-MB-468 showed the higher percentage of CSC, we decided to interrogate the effects of P2Et in vivo. Two groups of NSG mice were engrafted with MDA-MB-468 and treated twice a week with P2Et (IP: 18.7 mg/Kg) or PBS. (Fig. 6a). P2Et treatment delayed tumor growth compared with control group from 32 to 56 day. However, from day 60 these differences were lost, and the control group was euthanized on day 67 by endpoint criteria, while half of the mice from the P2Et group remained alive until day 87 they were finally euthanized (Fig. 6b). To note, P2Et changed the metastasis profile observed in both groups. First, the group treated with PBS had macro-metastasis in the opposite mammary gland, peritoneum, kidney, mesentery and intestine; while, the group treated with P2Et had macro-metastasis only in the peritoneum, kidney and intestine (Supplementary Fig. S9). With these results, we have shown that the P2Et extract has a direct effect on the primary TN tumor, significantly retarding tumor growth allowing significantly longer survival. Despite early activity on the tumor, this control was lost over the time; possibly due to the fact that these animals are immunodeficient, which does not let to assess the true role of the immune response in tumor control.

## Methods

### Natural products

*Caesalpinia spinosa* pods were collected in Villa de Leyva, Boyacá, Colombia and identified by Luis Carlos Jiménez at the Colombian National Herbarium (voucher specimen number COL 523714, Colombian Environmental Ministry agreement number 1470 related to the use of genetic resources and derived products). The P2Et extract was obtained from *Caesalpinia spinosa* as previously described^23^.

### Tissue acquisition and processing

All primary tumor samples (Table 1) were collected under informed consent from individuals being treated at the Hospital Universitario San Ignacio and Centro Javeriano de Oncología under a protocol approved by the ethics committees. Samples from healthy donors were collected from patients with benign breast pathologies (fibroadenoma) or reduction mammoplasty. Collected samples were minced into small pieces and digested with 2 mg/mL of collagenase (Worthington biochemical corporation, NJ, USA) and 100 U/mL of hyaluronidase (Sigma-Aldrich, St Louis, MO, USA) at 37 °C overnight to generate organoids, then cultured in modified M87 media^25^ for 24 h and then flow cytometry characterization or viability assays were performed.

### Tumour cell line and culture condition

The human breast cancer cell lines: MCF-7 (Invasive breast ductal carcinoma, Luminal A, was obtained from American Type Culture Collection, Manassas, VA, USA), MDA-MB-468 (Metastatic breast adenocarcinoma, Triple negative) and BT-549 (Invasive breast ductal carcinoma, Triple negative) were cultured as previously reported^23^, and were provided by Dr. Pedro Romero (Ludwig Center for Cancer Research, Department of Oncology, Faculty of Biology and Medicine University of Lausanne, Switzerland). Tumor cells were proven *Mycoplasma* free using a MycoProbe Mycoplasma Detection Kit (R&D Systems) and maintained with ciprofloxacin (0.5 μg/mL).

### Flow cytometry

The phenotype of cells from tissues or cells lines were assessed by flow cytometry using a FACSAria II instrument (BD Immunocytometry Systems, San José, CA, USA). CSC surface phenotype was interrogated by staining with anti-human CD45-APC/Cy7 (Biolegend San Diego, CA, USA #368516), anti-human CD31-PE (BD Biosciences, San Diego, CA, USA #555446), anti-human CD140b-APC (Biolegend San Diego, CA, USA #323608), anti-human CD24-FITC (BD Biosciences, San Diego, CA, USA #555427), anti-human/mouse CD44-PE/Cy7 (eBiosciences Inc, San Diego, CA USA #25-0441-82), anti-human/mouse CD49f-Brilliant violet 421 (Biolegend San Diego, CA, USA #313624) and anti-human EPCAM-PE (BD Biosciences, San Diego, CA, USA #347198). ALDH catalytic activity was evaluated with ALDEFLUOR assay kit (Stem Cell Technologies, Vancouver, BC, Canada) according to manufacturer indications. The antibodies anti-human MRP1-PE (Santa Cruz Biotechnology, Dallas, Tx, USA #sc-18836), anti-human CD338-Pacific Blue (Biolegend San Diego, CA, US #332012) and anti-human Pgp-FITC (BD Biosciences, San Diego, CA, USA #557002) were used to evaluate the drug efflux pumps expression. Finally, the results were analysed using FlowJo software V10 (Tree Star, Inc., Ashland, OR, USA).

To evaluate calreticulin (CRT) expression, 2 × 10^5^ cells were plated in 12-well plates and 12 h later cells were treated with P2Et (136.7 and 273.4 μg/ml), Doxorubicin (DX 0.48 and 0.96 μM, positive control for immunogenic cell death markers), or ethanol for 24 and 48 h. Cells were stained as previously reported^32^. The cells were incubated with the primary antibody (rabbit polyclonal antibody against calreticulin # 2907, Abcam, Cambridge, UK) followed by the Alexa488- conjugated monoclonal secondary antibody (Molecular Probes, Eugene, OR, Canada). Each sample was then processed using a FACSAria II (Becton–Dickinson, NJ, USA) and analyzed with FlowJo software (Tree Star, Inc., Ashland, OR, USA). In every case, LIVE/DEAD Fixable Aqua Dead Cell Stain was used to exclude dead cells (Life Technologies, NY, USA).

To evaluate ROS production, 2 × 10^5^ cells were plated in 6-well plates and 12 h later the cells were treated with P2Et (136.7 and 27.34 μg/ml), DX (0.48 and 0.096 μM, positive control for ROS production), ethanol or DMSO for 6, 12 and 24 h. Cells were stained with 1 μM of 2′,7′-Dichlorofluorescin diacetate (DCFDA) (D6883 Sigma-Aldrich, MO, USA) for 40 min at 37 °C, followed by Propidium Iodide (Sigma-Aldrich, MO, USA). Each sample was then processed using a FACSAria II (Becton–Dickinson, NJ, USA) and analyzed with FlowJo software (Tree Star, Inc., Ashland, OR, USA).

### RT-PCR

Tissues samples from patients were stabilized using RNA*Later* solution at − 80 °C and were stored until use (Invitrogen, CA, USA # AM7021). Total RNA of tissue samples was extracted using Rneasy Mini Kit according to the manufacturer’s instructions (Qiagen MD, USA) and the cells lines using Trizol (Life Technologies, NY, USA # 15596018). For GSTO-1 expression, MDA-MD-468 cells were plated in 6-well plates and 12 h later cells were treated with P2Et (136.7 μg/ml), DX (0.48 μM), ethanol or DMSO for 24, 48 and 72 h. Total RNA was extracted using Trizol (Life Technologies, NY, USA # 15596018). The quality and quantity of RNA were assessed with a NanoDrop spectrophotometer (NanoDrop Technologies, Waltham, MA USA). cDNA was synthesized with SuperScript III Reverse Transcriptase (Invitrogen, CA, USA # 18080044), following the manufacturer's instructions. For real-time PCR reaction, 600 ng of cDNA, DNA Master Plus SYBR Green I (Roche Applied Science, IN, USA) and 250 nM of forward and reverse primers were added in a total volume of 20 μL. The following primers were used: **ABCG2** (F: ACGAACGGATTAACAGGGTCA; R: GCATTTGCTGTGCTTGAGTCTA), **ABCB1 (**F: TGCGACAGGAGATAGGCTG; R: CAAAATCACAAGGGTTAGCTT), **ABCC1** (F: GGCTGCGGAAAGTCGTCCC; R: GCCCCCAGACAGGTTCACGC), **Oct3/4** (F: GGGTCTCTCTTTCTGTCCTTTC; R: TCATTCACCCATTCCCTGTTC), **Nanog** (F: TGAAATCTAAGAGGTGGCAGAA: R: CCTGGTGGTAGGAAGAGTAAAG), **Sox2** (F: GGAGAGTAAGAAACAGCATGGA; R: GTGGATGGGATTGGTGTTCT) and **GSTO-1** (F: GAACGGCTGGAAGCAATGAAG; R: TGCCATCCACAGTTTCAGTTT). Reactions were performed in triplicates using QuantStudio 3 Real-Time PCR systems (ThermoFisher Scientific, Waltham, MA, USA). The thermal cycling conditions were as follows: an initial denaturing step at 95 °C for 10 min, 40 cycles at 95 °C for 10 s, 60 °C for 10 s, and 72 °C for 10 s, followed by a dissociation stage. The relative expression levels of the ABCB1, ABCG2, ABCC1, OCT4, Nanog, SOX2 and GSTO-1 genes were normalized to the endogenous control gene β2-microglobuline. Relative expression was calculated using the comparative method or 2^−ΔCT^^33^.

### Three-dimensional primary tumor cultures and drug response assays

Tumor cells from solid tumors were grown in 3D cultures to form organoid structures. First, the single cells were suspended in modified M87 media and plated in a 24‐well ultra-low attachment tissue culture plate for 24 h at 37 °C in 5% CO_2_ to form organoid structures. Cells were then diluted in media and added to BD Matrigel Matrix Growth Factor Reduced (BD Biosciences) for a total volume of 1:1 matrix to media. A total of 100 μl was then seeded into a 96‐well assay plate ultra-low attachment overnight. Next, cells were incubated with P2Et (120, 30, 13.3 and 4.4 μg/mL), DX (3. 1.6, 0.5, 0.18 μM, conventional breast cancer treatment) or Ethanol (negative control) for additional 72 h. Viability was determined using LIVE/DEAD Viability/Cytotoxicity Kit *for mammalian cells* (Molecular probes, Eugene OR, USA #L3224) using Calcein AM (3 μM) and Ethidium homodimer-1 (4 μM) for live and dead cells, respectively^25^. Stained organoids were analyzed using a confocal microscope FV1000 (Olympus, Conklin, NY, USA). 488 nm line and 515 nm line from 30mW argon laser were used to stimulated calcein and ethidium homodimer-1 fluorescence, respectively. 640 × 640 images were acquired with a 10X UPLFLN, NA: 0.30 objective. Between 3 to 6 organoids per treatment were imaged and analyzed. Each image was analyzed using FIJI (ImageJ, version 1.51, NIH, USA). All images were equally processed adjusting contrast and saturation associated to each fluorescence channel. Area in terms of pixels was calculated for each separated fluorescence channel (Calcein and ethidium homodimer-1), thus the percentage of cell death (area from ethidium homodimer-1) respect to total area (areas from calcein + ethidium homodimer-1) was obtained.

### In vitro cytotoxicity assays

Cytotoxic effects on cell lines were evaluated using MTT (Sigma-Aldrich, Saint Louis, MO) and trypan blue dye assays. Cells (5 × 10^3^ cells/well) were seeded in 96-wells plates with ethanol (0.02%) as negative control, P2Et (250–0.95 μg/ml) or DX (5–0.03 μM) as positive control for 48 h. The cytotoxic effect was estimated by MTT assay according to procedure previously described^34^. The IC_50_ (50% inhibition of cell growth) value was calculated using a no linear regression log (inhibitor) versus response–variable slope graph in GraphPad Prism (GraphPad Prism 8 Software, La Jolla, CA, USA).

Synergistic effects were assessed over a dose–response matrix that included eight concentrations of P2Et (ranging from 0–500 μg/ml) and DX (0 to 1.6 μg/ml). The effects of drug combination were estimated using R Synergy Finder pipeline^35^ and the zero-interaction potency (ZIP) model^36^ was used to generate synergy score matrix from a dose–response matrix. At least two independent experiments with triplicate datasets were performed for each treatment.

### Proliferation assays

Single-cell suspensions of MDA-MB-468 were stained with 1 µM CFSE (Invitrogen, CA, USA) in PBS for 20 min at 37 °C, centrifuged, washed, and counted. 8 × 10^3^ CFSE-labeled tumor cells were seeded in 24-wells plates with ethanol (0.02%) as negative control, P2Et extract (IC_50_), DX (IC_50_) or P2Et + DX (IC_50_) for 48, 72 and 96 h. Samples were counted by trypan blue and then processed using a FACSAria II instrument (BD Immunocytometry Systems, San José, CA, USA) and proliferation was analyzed with FlowJo software V10 (Tree Star Inc, Ashland, OR, USA).

### Quantification of cell death

Cell death was quantified using Annexin V-Alexa Fluor 488 (Molecular Probes, Invitrogen Corp, Carlsbad, CA, USA) and propidium iodide (PI; Sigma), as previously reported^21^. Four independent experiments with duplicated samples were acquired on a FACSAria II (BD Immunocytometry Systems, San José, CA, USA) and analyzed with FlowJo software V10 (Tree Star Inc, Ashland, OR, USA).

### ATP release evaluation

MDA-MB-468 cells (2 × 10^5^, in 6 well-plates) were treated with vehicle (ethanol), P2Et (136.7 and 273.4 μg/ml), DX (0.48 and 0.96 μM, positive control for immunogenic cell death)**,** for 12 h. Cells were collected and stained as previously described^37^. Briefly, quinacrine was prepared to 1 μM final concentration in Krebs–Ringer solution. Cells were loaded with quinacrine warm solution for 30 min at 37 °C, washed and resuspended with 1 mg/ml of PI solution. Two independent experiments by duplicated samples were acquired on a FACSAria II (BD Immunocytometry Systems, San José, CA, USA) and analyzed with FlowJo software V10 (Tree Star Inc, Ashland, OR, USA).

### Animals and in vivo effect of P2Et in MDA-MB-468 BCTN model

NOD. Cg-Prkdc^*scid*^ IL2rg^tm1Wjl^/SzJ, most often known by their branded name, NOD *scid* gamma (abbreviated as NSG) mice (3–4 weeks old) were purchased from The Jackson Laboratory (Bar Harbor, ME). All mice were housed in a pathogen free facility in microisolator cages following the established protocols of the Ethics Committee of the Science Faculty and National and International Legislation for Live Animal Experimentation (Colombia Republic, Resolution 08430, 1993; National Academy of Sciences, 2010) (Act 006-2014, approved on June 20, 2014). Mice were housed in polyethylene cages with sterile food and water ad libitum, controlled temperature, and a 12 h light/dark cycle. Before treatment, the mice were familiarized for 1 week under standard conditions. The ethics committee of the science faculty approved the format for animals use on June 24, 2014.

1 × 10^6^ MDA-MB-468 cells were orthotopically transplanted on NSG mice. After 8 days, the mice (4 mice per group) were treated intraperitoneally with P2Et (18.7 mg/kg) or PBS twice per week, until endpoint. Tumours were measured with Vernier callipers twice a week, and the experiment was finished when mice showed end-point symptoms, humanely defined in the protocol. The tumour volume was calculated using the following formula: tumour volume (mm^3^) = [(Width)^2^ × Length]/2.

### Statistical analysis

Statistical analysis was performed with GraphPad Prism software version 8.0 (Graph Pad Prism Software Inc, San Diego, CA). Specific statistical tests were applied as explained in each figure legend. For breast cancer patients' samples and in *vitro* experiments, multiple comparisons were calculated by one-way ANOVA with Dunnet T3 correction or Tukey post-test analysis and unpaired t-test, and p value of < 0.05 was considered statistically significant. The specific statistical test results are indicated in each figure: *p < 0.05; **p < 0.01; ***p < 0.001; ****p < 0.001. Correlations were assessed using nonparametric Spearman correlation, determination coefficient r and *p*-value are shown. For in vivo experiments multiple t-test with Holm-Sidak method correction was performed assuming significance of α = 0.05.

## Discussion

Traditional Chinese Medicine (TCM) and particularly Phyto-therapy has been used for centuries to improve the response to treatment in cancer patients, and there is currently strong evidence about its positive effects in patient survival and improving the quality of life in response to chemotherapeutic or biological treatment^43^. The mechanisms involved in the activity of plant-derived drugs have been extensively studied, and it has been reported that they can modulate p53, decrease of oncogenic proteins activation or expression, induce cell differentiation via epigenetic modifications, and alters the tumor microenvironment and decreasing metastatic spread by targeting CSC^44,45^.

Among plant-based drugs, polyphenols, are a broad category of plant metabolites that has been implicated in cancer control. They are characterized for the presence of one or more benzene rings attached to hydroxyl groups. They have health benefits attributed to their antioxidant capacity, neuroprotective and anti-inflammatory effects^46^.

For some polyphenols, its antitumor activity and its effect on the reduction of metastases, are also related to the induction of autophagy by a non-canonical route, the decrease in the drug-efflux and the impairment of signaling pathways involved in self-renewal^37,47^. Additionally, its pro-oxidant effect at high concentrations may be involved in mitochondrial dysfunction and decreased cell proliferation^20,48^.

We have previously shown that P2Et extract, a polyphenol-rich extract, have a direct effect on murine melanoma and breast cancer tumors. Previous reports of P2Et, have shown that this extract is harmless in vivo and in vitro and do not affect T-cell activation in healthy mice^38^. We have also shown that P2Et induced mitochondrial-dependent apoptosis. Recently, we demonstrated that P2Et partially acts through a mechanism involving PERK-dependent endoplasmic reticulum stress and Ca2 + unbalance causing mitochondrial-dependent apoptosis^31^ . P2Et extract also induced immunogenic signals release such as calreticulin, ATP and HMGB1^21,22^, which explains the effects that partially relies on T-cell activation^21,22^ .

Furthermore, we demonstrated that P2Et impair stemness in ALDH^+^ cells (4T1-H17 cells), decreasing their sphere-formation ability and drug-resistance^23^ . Besides, the in vivo effects of P2Et against ALDH + enriched models required the activation of the immune response^24^.

To date, P2Et has not been tested in human models of cancer. Hence, the objective of this work was interrogating the activity of P2Et in organoids derived from human specimens and correlate it with the percentage of BCSC.

A large amount of evidence indicates that BCSCs drive tumor development and explains the regenerative capacity, dormancy, chemo-resistance, and metastatic ability^7^. BCSCs can be isolated from fresh surgical specimens, using the currently established stem cell surface markers EpCAM, CD44, CD49f (or CD29), CD24, and ALDH1^25^, and enriched ex vivo by their ability to form spheroids^25^. Here we isolated and studied BCSC of primary tumors from patients with breast cancer who received or not NAT before surgery. Our data indicated that luminal and TN patients have a higher frequency of intra-tumoral BCSC (Lin^-^CD44^+^CD24^−^CD49f^+^EPCAM^+^), which are enriched after NAT therapy and express ALDH^+^, BCRP1^+^ and Pgp^+^ but not MRP1^+^ as compared with mammary cells obtained from normal human donors. We also observed a positive correlation between BSCS markers and *ABCG2* gene or BCRP1 protein expression, which was also positively correlated with the expression of ALDH in the group of patients with NAT before surgery. As well, the group of patients that received NAT, a positive correlation between ALDH and *ABCC1* gene expression was observed. In fact, related to our findings, ALDH1 gene expression has also been shown to be higher in TNBC than in Luminal A, Luminal B and HER2 + subtypes^39^. Also, our results confirm previous observations showing increased CD44^+^/CD24^−^ and ALDH1 + cells in basal-like tumors compared to luminal A and B^40^, and reinforce the relationship between CSC and the expression drug resistance related markers.

Our results confirm previous work suggesting that BCSC, can be an important target and prognostic marker^41^. Previously, the lack of efficacy of NAT against the PIK3CA-defective BCSCs, one of the most commonly found genetic mutation in breast cancer, have been reported^42^. This means that NAT might select CSCs not only because of their intrinsic resistant to chemotherapy, but also because of the induction of factors that enhance tumor survival. In this sense, it has been observed that treating human or murine TNBC cells with chemotherapeutics, induces a coordinate transcriptional program of CD47, CD73, and PD-L1, leading to higher percentage of CD47^+^CD73^+^PDL1^+^ breast cancer cells. Moreover, chemotherapy induces the expression of GSTO1, which is dependent of HIF-1 and HIF-2, and knockdown of GSTO1 expression abrogates carboplatin-induced BCSC enrichment, decreasing tumor initiation and metastatic ability, and delaying tumor relapse^15,16^.

In our hand, P2Et and DX acts through different mechanisms, killing a TNBC cell line (MDA-MB-468) by decreasing intracellular ROS without an induction of GSTO1. Meanwhile, it has been reported that DX induce HIF activation^43^, yet, we do not have evidence of HIF activation by P2Et, supporting the fact that polyphenols have been related to a decrease HIF activation^43,44^. For the first time, we observed that P2Et disrupt the organoids derived from human patients, and these effects were improved in combination with standard chemotherapy (DX). Previously, we observed similar synergistic effects in vitro, in a mechanism involving pump efflux inhibition^23^. However, additional effects that could induce collateral sensitivity cannot be discarded^45^.

These results suggested that drug-resistance induced by NAT in TNBC, could be partially overcome by P2Et used as adjuvant. This hypothesis is based on the evidence that P2Et targets stemness^23^, induced immunogenic cell death^21,22,31^ that triggers a T-cell immune response^22,24^ and it is cytotoxic to 3D organoids derived from patients^46^.

In order to further evaluate in vivo the impact of P2Et in BCSC-enriched breast cancer models (triple negative and luminal), we analyzed the expression of CD44, CD24, EpCAM, CD49f and ALDH in human cell lines. Our results showed that MDA-MB-468 cells contained the highest frequency of BCSC (Supplementary Figs. S1c,d and S5a). Also, MDA-MB-468 cells had higher ALDH activity compared to BT-549, and it was according to previous reports that used the same cell lines^47,48^ .

Targeting ALDH + BCSC is important for several reasons. First, because ALDH is an important marker of drug resistance as mentioned above^49^, and second, because the peptides from the intracellular processing of ALDH can be presented in the context of MHC, unleashing BCSC-specific cytotoxic T-cell^50^.

In light of this, we implanted the MDA-MB-468 cells into NSG mice and we administered P2Et. We observed a reduction in tumor growth and less metastatic spread, however the differences in median survival were not significant, this support the fact that P2Et need an intact immune system to exert a better control of the disease, as we previously showed^24^; we are aware that the role of innate and immune system in humanized models must be tested in the future^24^.

In summary, P2Et has the ability to act on BCSCs, increasing their sensitivity to chemotherapy, and inducing signals that can lead the activation of an immune response, needed for tumor control. These hypotheses must be evaluated in clinical trials with cancer patients and other models that allows to interrogate the role of human immune system in the control of tumor and metastasis upon P2Et and chemotherapy combination.

### Ethcs approval and consent to participate

All participants provided informed consent and the study was approved by the ethics committee of the Science Faculty, Pontificia Universidad Javeriana, (Approved on June 19, 2014, Act Number 10-2014). All of the methods were performed in accordance with the Declaration of Helsinki and the relevant guidelines. All animal studies were approved by the Institutional Committee for the Care and Use of Laboratory Animals of the Pontificia Universidad Javeriana (CICUAL, PUJ, FUA 013-14, Approved on June 20/2014, Act Number 006-2014).

## Supplementary information

Supplementary Information.

## Data Availability

The datasets generated and/or analyzed during the current study are not publicly available as the informed consent does not cover such release and, further, in compliance with current data protection regulations. Contingent on ethical and data protection board approval, the access to the data are available from the corresponding author on reasonable request.
