# Supplementary material for: Evaluation of chemotherapy and P2Et extract combination in ex-vivo derived tumor mammospheres from breast cancer patients
Source: Sci Rep. 2020 Nov 12;10:19639. doi: 10.1038/s41598-020-76619-9 (PMC7665196; doi:10.1038/s41598-020-76619-9)
Supplement: Supplementary file 1 — Supplementary Information. [file 41598_2020_76619_MOESM1_ESM.docx]

**Evaluation of chemotherapy and P2Et extract combination in ex-vivo derived tumor mammospheres from breast cancer patients**

Claudia Urueña^1*^, Tito A Sandoval^1^, Paola Lasso^1,^, Tawil M^2^, Alfonso Barreto^1^, Torregrosa L^2^, Susana Fiorentino^1*^.

^1^ Grupo de Inmunobiología y Biología Celular, Unidad de Investigación en Ciencias Biomédicas, Facultad de Ciencias, Pontificia Universidad Javeriana. Bogotá, Colombia. ^2^ Hospital Universitario San Ignacio, Centro Javeriano de Oncología, Facultad de Medicina, Pontificia Universidad Javeriana, Bogotá, Colombia.

* Corresponding Author: Susana Fiorentino, and Claudia Urueña. Grupo de Inmunobiología y Biología Celular, Pontificia Universidad Javeriana, 057-1-3208320 Ext 4025, Fax 4021, Carrera 7a. No. 43-82, Ed. 50, Lab. 101, Bogotá C.P. 110211, Colombia. email: [susana.fiorentino@javeriana.edu.co](mailto:susana.fiorentino@javeriana.edu.co), [curuena@javeriana.edu.co](mailto:curuena@javeriana.edu.co)

**Supplementary Information**

**
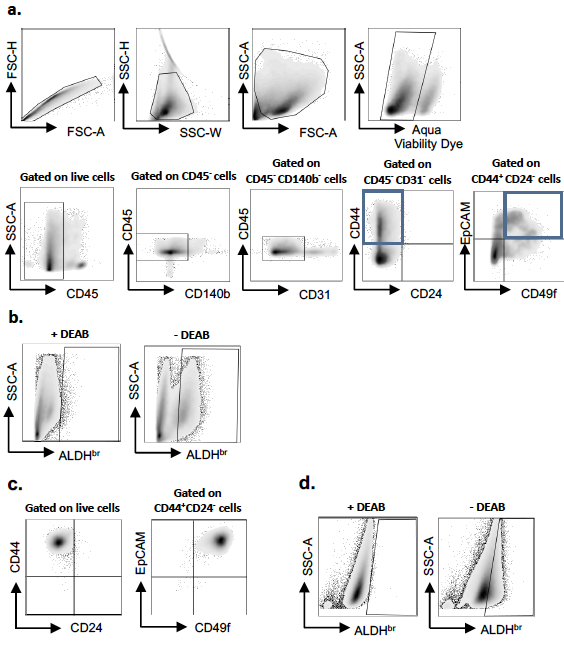
**

**Supplementary Figure 1. Representative flow cytometry plots illustrating gating strategy used to identify the viable breast cancer stem cells in tumor from patients and cells lines. a, b.** Gating strategy to identify BSCS markers in tumor from patients. **c, d.** Gating strategy to identify BSCS markers in breast cancer cell lines.

**
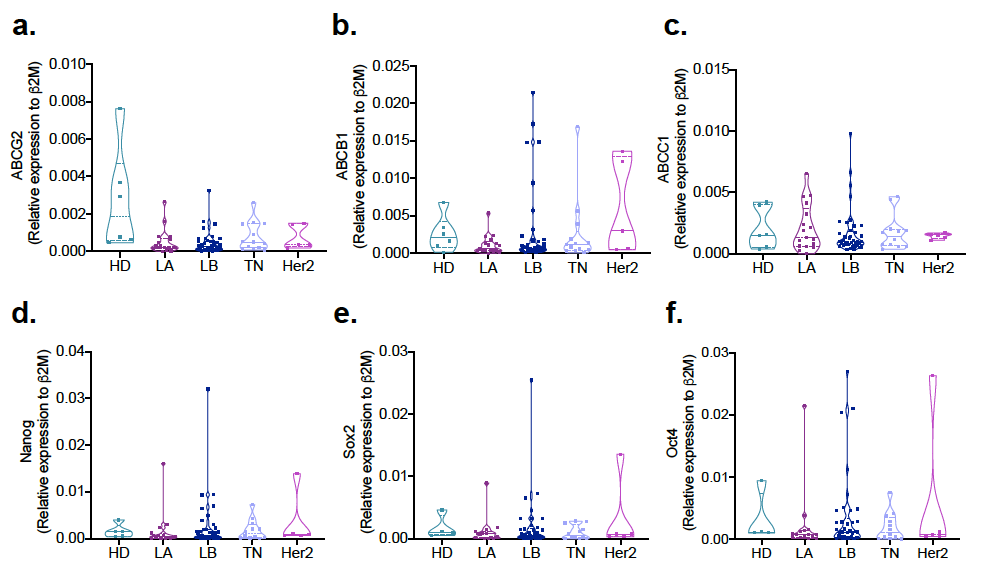
**

**Supplementary Figure 2.** Gene expression analysis related to cancer stem cells. Relative expression of the ABC pumps. **a.** ABCG2, **b.** ABCB1, **c.** ABCC1 and relative expression of the stem cell transcription factors. **d.** Nanog, **e.** Sox2, **f.** Oct4 by qRT-PCR in Healthy Donor (HD), Luminal A (LA), Luminal B (LB), Triple negative (TN) and Her-2. Data are presented as violin plots and each point represent independent sample.


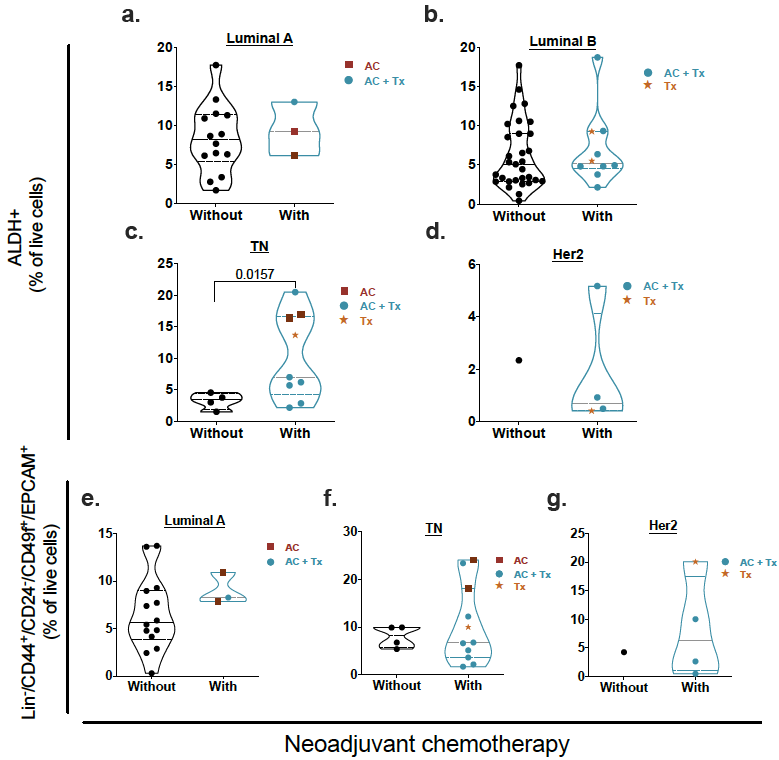


**Supplementary Figure 3. Frequency of BCSC ALDH+ and (Lin-/CD44+CD24-CD49f+EPCAM+) in breast cancer patients.** Frequency of BCSC (ALDH+) in patients who received (With) or not (Without) NAT before surgery in **a.** Luminal A **b.** Luminal B **c.** Triple Negative and **d.** Her-2/Neu. Frequency of BCSC (Lin-/CD44+CD24-CD49f+EPCAM+) in patients with or without NAT before surgery in **e.** Luminal A **f.** Triple Negative and **g.** Her-2/Neu. Data are presented as violin plots and each point represent independent sample. AC (Anthracyclines + Ciclophosphamide), AC+TX (Anthracyclines/Ciclophosphamide + Taxanes), TX (Taxanes). **p < 0.01


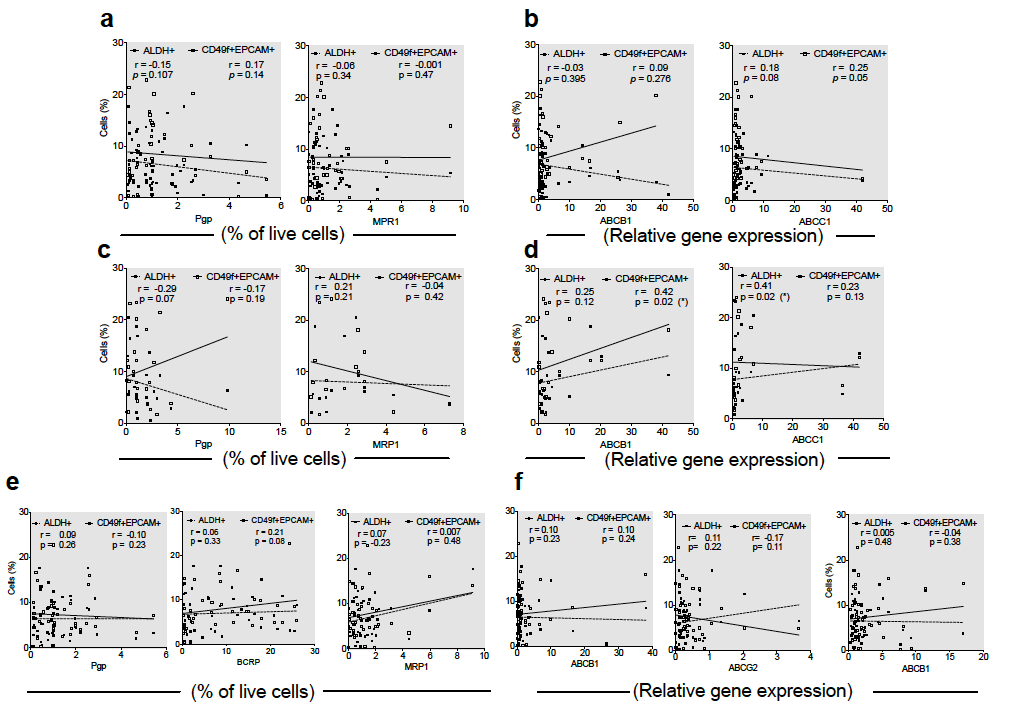


**Supplementary Figure 4. Correlation of multidrug efflux pumps expression with breast cancer stem cells markers in breast cancer patients.** Correlation of BCSC frequency (ALDH+ and CD24^-^CD44^+^CD49f^+^EPCAM^+^) in breast cancer patients **a.** Correlation with Pgp and MRP1 protein in all breast cancer patients. **b.** Correlation with *ABCB1* and *ABCC1* gene in all breast cancer patients. **c.** Correlation with Pgp and MRP1 protein in patients who received neoadjuvant chemotherapy before surgery. **d.** Correlation with ABCB1 and ABCB1 gene in patients who received neoadjuvant chemotherapy before surgery. **e.** Correlation with Pgp, BCRP and MRP1 protein expression in all breast cancer patients who did not received neoadjuvant chemotherapy before surgery. **f.** Correlation with *ABCB1*, *ABCG2* and *ABCC1* relative gene expression in all breast cancer patients who did not received neoadjuvant chemotherapy before surgery. Correlations were assessed using nonparametric Spearman correlation, determination coefficient r and *p*-value are shown. *p < 0.05.


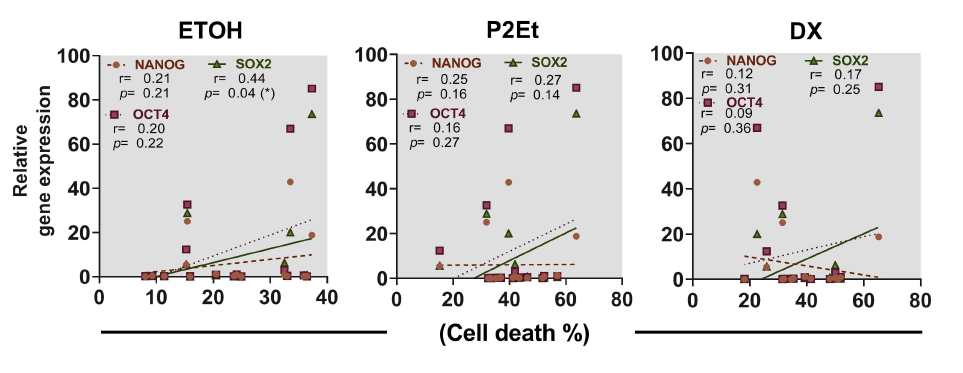


**Supplementary Figure 5. Correlation between cell death percentage after treatments with multidrug resistants pump genes.** Correlation between cell death percentage of mammospheres from breast cancer patients after treatment with Ethanol (negative control), P2Et or Doxorubicin and *ABCB1*, *ABCG2*, *ABCC1* gene expression. Correlations were assessed using nonparametric Spearman correlation, determination coefficient r and *p*-value are shown. *p < 0.05.


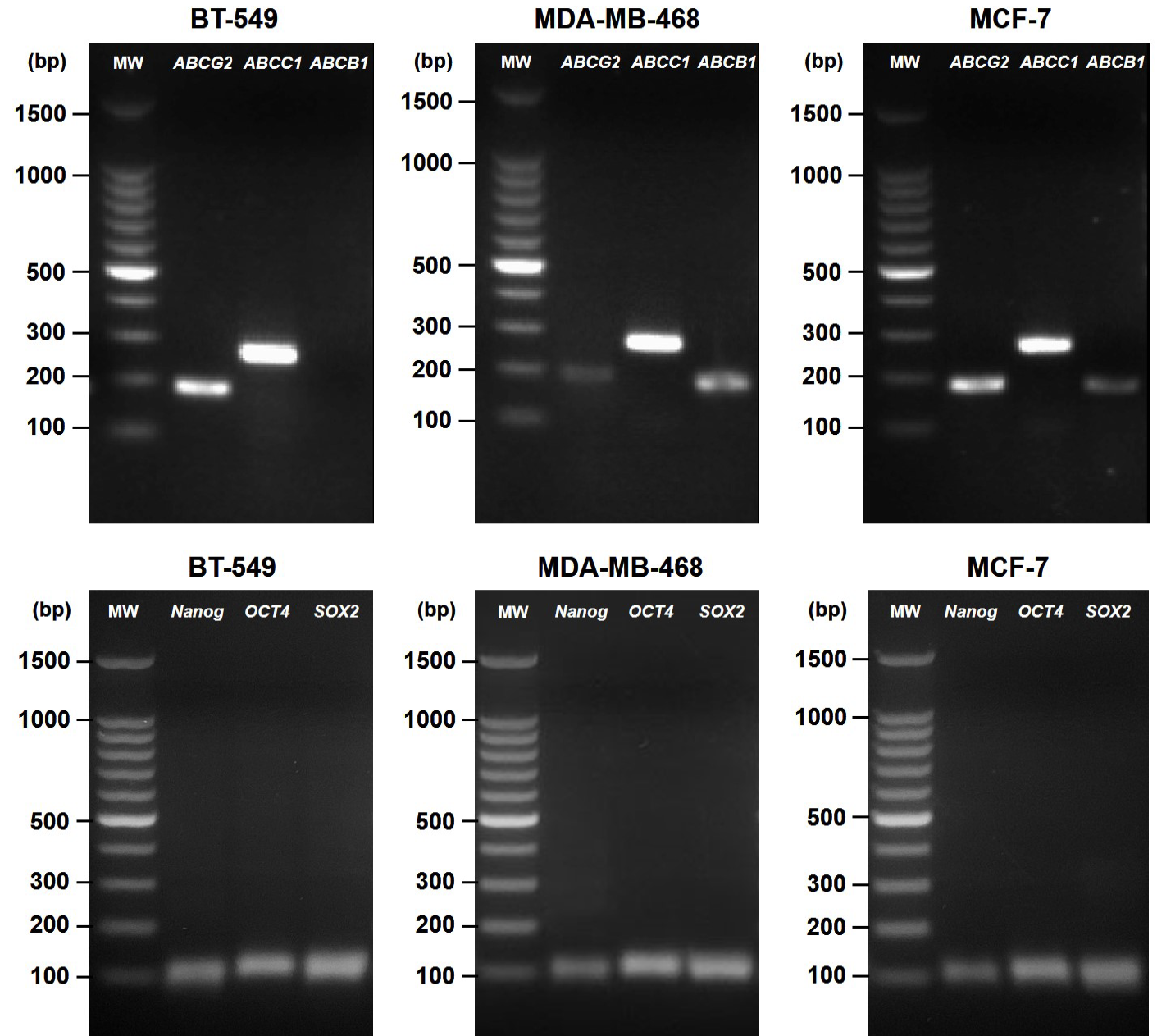


**Supplementary Figure 6. Gene expression of multi-drug resistance pumps and stem cell transcription factors. a.** Gene expression of ABCG2 (Pgp), ABCB1 (BCRP) and ABCB1 (MRP1) and **b.** gene expression of Nanog, OCT4 and SOX2 evaluated in BT-549, MDA-MB-468 and MCF-7 breast cancer human cells by RT-PCR.


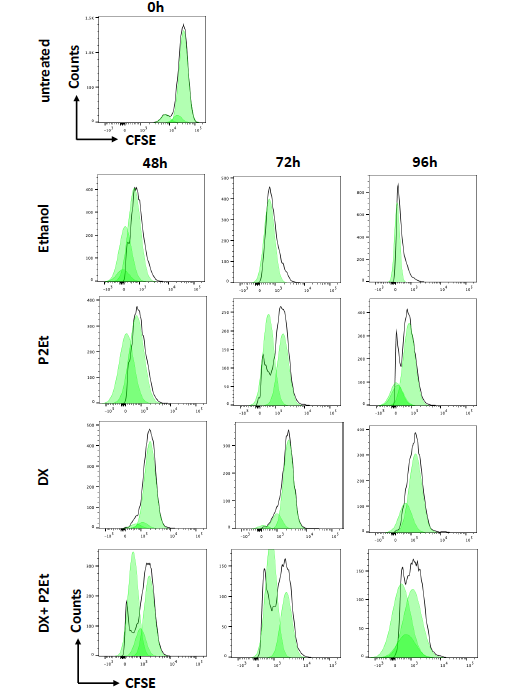


**Supplementary Figure 7. Representative histograms showing proliferation pattern of breast cancer cell line.** Proliferation pattern of MDA-MB-468 by CFSE after treatment with Ethanol (negative control), P2Et extract, Doxorubicine (DX) or P2Et + DX after 48, 72 and 96h. The proliferation analysis was performed using the proliferation tool of the FlowJo Software V10.7.1.


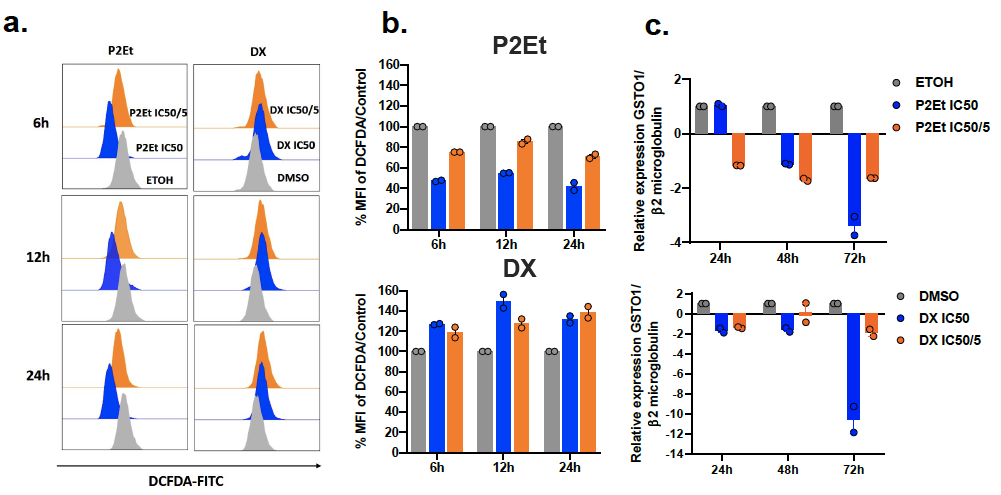


**Supplementary Figure 8. P2Et extract decrease ROS production and GSTO-1 gene expression.** MDA-MB-468 cell line were treated with P2Et extract or DX (IC_50_ and IC_50/5_) for 6, 12 and 24h. ROS production was evaluated using 2′,7′-Dichlorofluorescin diacetate (DCFDA) by flow cytometry. **a**. Representative histogram of mean fluorescence intensity (MFI) at 6, 12 and 24h; **b**. Frequency of MFI DCFDA/control cells by Flow cytometry after treatment with P2Et extract (upper graph) and DX (bottom graph); MDA-MB-468 cell line were treated with P2Et Extract or DX (IC_50_ and IC_50/5_) for 24, 48 and 72h, and **c**. Relative expression of GSTO-1 gene evaluated by qRT-PCR.


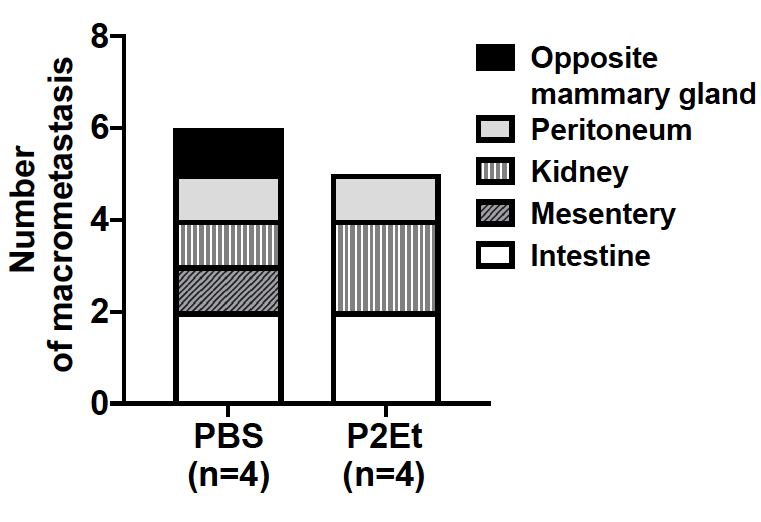


**Supplementary Figure 9. Number of macro-metastasis in mice treated with PBS or P2Et in triple negative cells.** Different organs with macro-metastasis and the number of mice that presented macro-metastasis between groups.
